# Selective tau vulnerability in the human master circadian clock in atypical Alzheimer's disease

**DOI:** 10.1002/alz70855_097326

**Published:** 2025-12-23

**Authors:** Gowoon Son, Mihovil Mladinov, Felipe Luiz Pereira, Yumi Yang, Grace Judge, Song Hua Li, Chia‐Ling Tu, Claudia Kimie Suemoto, Renata Elaine Paraizo Leite, Vitor Ribeiro Paes, Carlos Augusto Pasqualucci, Wilson Jacob‐Filho, Salvatore Spina, William W. Seeley, Wenhan Chang, Thomas C. Neylan, Lea T. Grinberg

**Affiliations:** ^1^ Memory and Aging Center, UCSF Weill Institute for Neurosciences, University of California, San Francisco, San Francisco, CA, USA; ^2^ Memory and Aging Center, UCSF Weill Institute for Neurosciences, University of California San Francisco, San Francisco, CA, USA; ^3^ San Francisco VA Medical Center, University of California San Francisco, San Francisco, CA, USA; ^4^ Division of Geriatrics, University of São Paulo Medical School, São Paulo, São Paulo, Brazil; ^5^ Physiopathology in Aging Laboratory (LIM‐22), Department of Internal Medicine, University of São Paulo Medical School, São Paulo, São Paulo, Brazil; ^6^ University of Sao Paulo Medical School, São Paulo, Brazil; ^7^ Division of Geriatrics, Department of Internal Medicine, University of São Paulo Medical School, São Paulo, São Paulo, Brazil; ^8^ Department of Neurology, Memory and Aging Center, University of California San Francisco, San Francisco, CA, USA; ^9^ Department of Psychiatry and Behavioral Sciences, University of California San Francisco, San Francisco, CA, USA

## Abstract

**Background:**

Recently recognized syndromic variants in cases with AD pathology reflect regional differences in susceptibility to AD‐tau pathology. We found systematic differences in sleep architectures between amnestic (amAD) and atypical AD (atypAD), including evidence of a more blunted circadian rhythm pattern in amAD compared to atypAD. However, the underlying neurobiological basis remains unknown. We hypothesize that amAD cases exhibit greater degeneration of the suprachiasmatic nucleus (SCN) than atypAD. The link between increased regional susceptibility in AD variant and sleep physiology may be involved in protein dysregulation, contributing to AD pathololgy.

**Method:**

We analyzed 27 postmortem SCN tissues from controls without neuropathological change and individuals with amnestic AD or atypical (logopenic) AD. We quantified arginine vasopressin (AVP+) and vasoactive intestinal peptide (VIP+) positive neurons and conducted semi‐quantitative counts of neurofibrillary tau. Moreover, we applied in‐situ proteomics using GeoMx Digital Spatial Profiler (DSP) to examine expression levels of 57 proteins commonly dysregulated in amnestic AD.

**Result:**

AtypAD exhibited lower tau burden and higher SCN neuronal counts. The differences between AD variants was more pronouced in AVP+ neurons. Amyloid pathology was absent in all cases. Spatial proteomics analyses revealed that AD‐associated proteins showed more preserved pathoprotein expression in atypAD than amAD.

**Conclusion:**

Our findings suggest that the SCN is more severely affected in amAD than in atypAD, exhibiting greater tau burden and neuronal loss, particularly in AVP+ neurons. This supports the hypothesis that differences in regional vulnerability to AD‐tau pathology contribute to AD variant phenotypes and may underlie the blunted circadian rhythms observed in amAD. Additionally, spatial proteomics revealed more preserved pathoprotein expression in atypAD, suggesting distinct molecular dysfunctions across AD variants. These findings highlight the need for further investigation into molecular factors driving protein dysregulation, such as neuroinflammatory or lysosomal disruptions, as key contributors to differential susceptibility in AD pathology.

